# Diagnostic Performance of a Panel of miRNAs (OsteomiR) for Osteoporosis in a Cohort of Postmenopausal Women

**DOI:** 10.1007/s00223-020-00802-3

**Published:** 2021-01-11

**Authors:** K. Kerschan-Schindl, M. Hackl, E. Boschitsch, U. Föger-Samwald, O. Nägele, S. Skalicky, M. Weigl, J. Grillari, P. Pietschmann

**Affiliations:** 1 Department of Physical Medicine, Rehabilitation and Occupational Medicine, Medical University of Vienna, Vienna, Austria; 2 TAmiRNA GmbH, Vienna, Austria; 3 Austrian Cluster for Tissue Regeneration, Vienna, Austria; 4 KLIMAX Menopause and Osteoporosis Clinic, Vienna, Austria; 5 Institute of Pathophysiology and Allergy Research, Center for Pathophysiology, Infectiology and Immunology, Medical University of Vienna, Vienna, Austria; 6 Christian Doppler Laboratory for Biotechnology of Skin Aging, Department of Biotechnology, BOKU - University of Natural Resources and Life Sciences Vienna, Vienna, Austria; 7 Ludwig Boltzmann Institute for Experimental and Clinical Traumatology, 1220 Vienna, Austria

**Keywords:** Postmenopausal osteoporosis, Fragility fracture: circulating microRNA, osteomiR, Bone biomarker

## Abstract

A specific signature of 19 circulating miRNAs (osteomiRs) has been reported to be associated with fragility fractures due to postmenopausal osteoporosis. However, it is unknown whether osteoporotic fractures or low BMD phenotypes are independently contributing to changes in osteomiR serum levels. The first aim was to characterize the abundance, sensitivity to hemolysis, and correlation of osteomiR serum levels, the second objective to evaluate the diagnostic accuracy of osteomiRs for osteoporosis according to the WHO criteria and on basis of major osteoporotic fracture history. Fifty postmenopausal women with osteoporosis (with or without fragility fracture) and 50 non-osteoporotic women were included in this cross-sectional study. The diagnostic performance of osteomiRs for osteoporosis based on the WHO definition or fracture history was evaluated using multiple logistic regression and receiver-operator curve (AUC) analysis. The osteomiR® signature is composed of four clusters of miRNAs providing good performance for the diagnosis of osteoporosis in postmenopausal women defined by WHO criteria (AUC = 0.830) and based on history of major osteoporotic fractures (AUC = 0.834). The classification performance for the WHO criteria and for fracture risk is driven by miR-375 and miR-203a, respectively. OsteomiRs, a signature of 19 emerging miRNA bone biomarkers, are measurable in human serum samples. They constitute a panel of independent bone and muscle biomarkers, which in combination could serve as diagnostic biomarkers for osteoporosis in postmenopausal women.

**Supplementary Information:**

The online version of this article (doi:10.1007/s00223-020-00802-3) contains supplementary material, which is available to authorized users.

## Introduction

Osteoporosis causes worldwide approximately 1000 fractures per hour and about 9 million fractures annually [1]. Besides an enormous economic burden osteoporosis has been linked with a loss in quality of life. Currently, bone mineral density (BMD) measurement by dual energy X-ray absorptiometry is the gold standard to diagnose osteoporosis. However, areal BMD only poorly identifies elderly women and men at high fragility fracture risk [2]. The bone turnover markers (BTMs) C-terminal telopeptide of type I collagen (CTX) and procollagen type I aminoterminal propeptide (P1NP) are frequently used in clinical routine. However, as already shown these BTMs have not proved to be associated with hip fracture risk [3].

The consequence of the limited discriminating power of areal BMD and BTMs in evaluating subjects at high and low fracture risk leads to the missed opportunity of fracture prevention. Research evidence suggests that circulating microRNAs give valuable information concerning bone status and may serve as biomarkers for bone diseases [4]. MicroRNAs (miRNAs) are small, non-coding RNAs of approximately 20 to 25 nucleotides in length that post-transcriptionally regulate gene expression. MiRNAs were demonstrated to be involved in various biological processes including the differentiation, proliferation, and apoptosis of bone cells (for review see [5]) as well as cellular senescence [6].

Several studies have shown that some miRNAs are increased, whereas the serum level of other miRNAs appears to be reduced in patients with osteoporosis compared to non-osteoporotic controls [7–10]. A series of publications has also discovered different circulating miRNAs to be altered in patients with osteoporotic fractures [11–17]. Specific combinations of miRNAs have been reported to be associated with the risk of fragility fractures in postmenopausal osteoporosis [13, 15]. The high sensitivity and specificity (85%) of osteomiR—a panel of miRNAs—in serum makes the ability of osteomiR to identify subjects at high risk of fragility fracture very likely [16]. However, previous studies have mostly applied miRNA screening assays to assess a large number of miRNAs in relatively small cohorts, resulting in selection of the osteomiR miRNA panel, and only two studies have aimed to further assess this specific miRNA panel in further independent cohorts. Zarecki et al. [18] have individually analyzed osteomiRs in three groups of postmenopausal women: vertebral fractures, low BMD without fractures, and controls. They observed that the presence of vertebral fractures had a stronger impact on osteomiR levels compared to low BMD. Ladang et al. [19] have performed a pilot study to evaluate the prognostic value of osteomiRs for fracture-risk assessment using combined evaluation based on a multivariate model.

In order to validate the clinical utility of osteomiRs as bone biomarkers, and to improve our understanding of pre-analytical factors that bias osteomiR analysis in clinical routine, further studies taking advantage of larger cohorts of postmenopausal women must be performed. Therefore, the first objective of this cross-sectional study was to characterize the abundance, sensitivity to hemolysis, and correlation of osteomiR levels in serum samples of postmenopausal women, since this had not yet been reported. The second objective was to assess the combined diagnostic performance of osteomiR based on two differing stratification criteria—bone mineral density and fragility fracture—in 100 subjects attending an outpatient clinic for evaluation of bone status.

## Material and Methods

### Study Design

This was a single center cross-sectional study including patients presenting at the menopause and osteoporosis outpatient clinic Klimax in Vienna. The study protocol was approved by the ethical committee of the Medical University of Vienna (Approval Number 1463/2014) and performed in accordance with the ethical standards of the 1964 Declaration of Helsinki and its subsequent amendments. All participants provided their written informed consent after the procedure of the trial had been explained to them.

### Subjects

A total number of 100 postmenopausal women were recruited from the Austrian menopause and osteoporosis outpatient clinic. Women were referred to the clinic either to prevent or treat the climacteric syndrome and other endocrine disorders, particularly postmenopausal osteoporosis. General characteristics of the women attending this clinic and the procedures of the institution were described by Boschitsch et al. [20] The women were divided into two groups according to the following criteria: the first group of women (osteoporotic women) consisted of subjects with either a T score (measured at the lumbar spine and/or the femoral neck) at or below − 2.5 SD or a fragility fracture in the presence of a *T* score at or below − 1.0 SD. The second group of subjects (non-osteoporotic women) comprised women free of fragility fractures with normal bone mineral density (BMD) or osteopenia (according to the definition of the World Health Organisation (WHO) measured at the lumbar spine and/or the femoral neck. Two subjects who presented with the history of a fracture and normal BMD were classified as non-osteoporotic women because their fractures were categorized as non-osteoporotic.

To be eligible, participants had to be at least 50 years of age. Exclusion criteria were secondary reasons for osteoporosis including inflammatory diseases, non-osteoporotic bone disease, severe renal or hepatic insufficiency, cancer within the previous 5 years, immobilization, and the intake of drugs with potential effects on BMD within the last three months (in the case of parenterally administered bisphosphonates or denosumab the exclusion period was extended to one year) and recent fractures (within 6 months). For subjects of the non-osteoporotic group, the history of a fragility fracture was defined as an additional exclusion criterion. All patients who fulfilled the criteria were included consecutively.

Besides the group assignment according to the WHO-based definition an additional assignment according to fracture-based classification was performed in our study subjects. One group compromised subjects with and one group subjects without a history of major osteoporotic fractures (MOS Fx). Fractures of the spine, hip, forearm, or humerus in the presence of osteoporosis or osteopenia were classified as major osteoporotic fractures [21].

### Study Procedures

In all subjects, medical history was obtained and physical examination was performed. A venous blood sample for the determination of routine blood chemistry, BTMs, and microRNAs was drawn after an overnight fast. Blood was subsequently allowed to clot. Thereafter it was centrifuged for 10 min with 2500 rpm; aliquots were stored at – 70 °C until analysis. BMD was measured at the lumbar spine and hip region. Based on clinical risk factors and BMD, the risk of major osteoporotic fractures and that of hip fractures was calculated by the FRAX™tool [21].

### Biochemistry

Regular biochemical analyses were performed in everyday practice and included the evaluation of serum calcium, phosphate, creatinine, gamma glutamyl transferase, C-reactive protein, parathyroid hormone, 25-OH-vitamin D, and alkaline phosphatase. All analyses were conducted according to routine procedures. Two bone turnover markers were also studied: osteocalcin (Oc; Cobas 8000 Analyzer, Roche Diagnostics, Switzerland, detection limit: 0.01 ng/mL; intra-assay coefficient of variation: 0.9–1.3%, inter-assay coefficient of variation: 1.2–2.3%) and cross-linked-C-telopeptide of type I collagen (CTX; Cobas 8000 Roche Analyzer, Roche Diagnostics, Switzerland, detection limit: 0.5 ng/mL; intra-assay coefficient of variation: 1.2–4.7%, inter-assay coefficient of variation: 1.5–5.7%).

### MicroRNA Analysis

MicroRNA analysis was performed by reverse-transcription quantitative PCR (RT-qPCR) using the osteomiR® RUO assay (TAmiRNA GmbH) according to the manufacturer’s instructions. Briefly, serum aliquots were thawed at room temperature, centrifuged at 12,000×*g* for 5 min to separate serum from any cellular debris or precipitate in the sample. For RNA extraction using the serum/plasma RNA extraction kit, 200 µl of serum was used. Synthetic RNA spike-in was added to the lysis buffer. Subsequently, RNA precipitation with 100% isopropanol was enhanced through addition of glycogen to the aqueous phase after extraction (final concentration 50 µg/ml). Total RNA was eluted in 30 µl nuclease-free water and stored at – 80 °C until further processing.

Reverse transcription was performed using the osteomiR® chemistry kit and 2 µl of total RNA. Following reverse transcription at 42 °C for 60 min and inactivation at 95 °C for 5 min, cDNA was stored at – 20 °C until PCR amplification.

PCR amplification was performed using components of the osteomiR® chemistry kit (miGreen mastermix) and the osteomiR® 384-well plates for Roche LC 480 instruments. PCR reactions were performed in 10 µl volume consisting of 5 µl cDNA (1:20 dilution) and 5 µl 2 × miGreen mastermix. The recommended 2-step protocol (45 cycles of 95 °C/10 s and 60 °C/60 s) followed by melting curve analysis (55–99 °C). Cq-values were calculated using the second-derivative maximum method implemented in the osteomiR® web-based analysis tool (https://osteomir.tamirna.com/). Normalization was performed based on RNA spike-in control using the following equation: Normalized delta Cq-value (∆Cq) = Cq^RNA Spike−In^ − Cq^microRNA^.

Quality control was performed by visual inspection of spike-in controls. Hemolysis was assessed based on the ratio of miR-23a/451a using the equation: hemolysis ratio (∆Cq) = Cq^miR−23a^ − Cq^miR−451a^.

### Bone Mineral Density Measurement

BMD was measured at the lumbar spine and the proximal femur by dual energy X-ray absorptiometry (DXA) using the same DXA device for all patients (Prodigy (fan beam) machine of the Lunar series; General Electrics Healthcare, Munich, Germany). Daily before operation, the device was calibrated according to the manufacturer’s standards and additionally a spine phantom was scanned to monitor performance.

### Statistical Analyses

Correlation analysis was performed in Graphpad Prism v8.3 using Pearson correlation and two-tailed tests for analysis of significance (*r* different from 0).

Unsupervised clustering analysis was performed in R/Bioconductor using the “dist” and “hclust” functions, Euclidean distance, and ward.D2 clustering.

One-way ANOVA analysis with post hoc tests was used to test for effects of increasing levels of hemolysis on osteomiR serum levels.

Multiple logistic regression analysis was performed in Medcalc v18.11.6.

Clustvis [22] was used to generate heatmaps for hemolysis expression data. Normalized dCq values were uploaded and unit variance scaling was applied to rows. No imputation was used since no missing values were present in the data. Both rows and columns were clustered using correlation distance and average linkage.

## Results

### Clinical Characteristics and Biochemical Parameters

Characteristics and biochemical parameters of osteoporotic and non-osteoporotic study participants are given in Table 1. Subjects with osteoporosis had the same age and a similar BMI as those with osteopenia/normal BMD. As expected, BMD levels were lower and FRAX scores were higher in osteoporotic than non-osteoporotic subjects; BTMs were significantly elevated as well. Routine clinical chemistry was similar in both groups.

**Table 1:** Characteristics and biochemical parameters: WHO classification

	Osteoporotic women (*n* = 50)	Non-osteoporotic women (*n* = 49)	*p*
Age (a)	67 [61; 76]	67 [61; 74]	n.s.
BMI	23.7 [20.9; 28.1]	24.8 [22.2; 27.4]	n.s.
*T* score femoral neck	− 1.7 [− 2.2; − 1.2]	− 0.9 [− 1.6; − 0.2]	< 0.0001
*T* score hip	− 1.6 [− 2.3; − 1.0]	− 0.8 [− 1.4; − 0.1]	< 0.0001
*T* score lumbar spine	− 2.6 [− 2.8; − 2.4]	− 0.8 [− 1.5; 0.3]	< 0.0001
FRAX major fracture	12.2 [8.6; 18.8]	8.6 [6.1; 12.0]	0.0002
FRAX hip fracture	3.1 [1.7; 5.7]	1.5 [0.7; 2.7]	< 0.0001
Calcium (mmol/l)	2.4 [2.4; 2.5]	2.4 [2.4; 2.5]	n.s.
Phosphate (mmol/l)	1.2 [1.1; 1.3]	1.2 [1.1; 1.3]	n.s.
Creatinine (mg/dl)	0.8 [0.7; 0.9]	0.8 [0.7; 0.9]	n.s.
GGT (U/l)	18 [12; 24]	18 [15; 26]	n.s.
C-reactive protein	0.14 [0.07; 0.23]	0.16 [0.07; 0.30]	n.s.
25-OH-vitamin D (mmol/l)	26 [19; 32]	24 [18; 33]	n.s.
PTH (pg/ml)	39 [31; 44]	39 [26; 52]	n.s.
AP (U/l)	74 [59; 86]	68 [59; 83]	n.s.
Osteocalcin (ng/ml)	22.4 [17.0; 26.2]	18.3 [16.2; 22.0]	< 0.05
CTX (ng/ml)	0.30 [0.22; 0.43]	0.24 [0.17; 0.31]	< 0.05

*GGT* gamma glutamyl transferase, *PTH* parathyroid hormone, *AP* alkaline phosphatase, *CTX* cross-linked-C-telopeptide of type I collagen

Table 2 shows the clinical characteristics and biochemical parameters of the study participants per fracture history (major osteoporotic fracture versus no major osteoporotic fracture). The *T* scores of all measured areas were lower and the FRAX scores were significantly higher for subjects with a history of a major osteoporotic fracture. Apart from a slightly different serum level of creatinine, no differences in biochemical parameters could be detected.

**Table 2:** Characteristics and biochemical parameters: MOFx classification

	MOFx (*n* = 18)	No MOFx (*n* = 81)	*p*
Age (a)	67 [61; 77]	67 [61; 74]	n.s.
BMI	25.8 [21.1; 31.2]	23.9 [21.2; 27.4]	n.s.
T score femoral neck	− 1.7 [− 2.3; − 1.4]	− 1.3 [− 1.7; − 0.4]	< 0.005
T score hip	− 1.9 [− 2.4; − 1.2]	− 1.0 [− 1.6; − 0.4]	0.0005
T score lumbar spine	− 2.6 [− 2.9; − 1.5]	− 1.5 [− 2.5;− 0.6]	< 0.0005
FRAX major fracture	17.0 [12.2; 23.9]	8.9 [6.3; 12.1]	< 0.0001
FRAX hip fracture	5.0 [2.8; 9.2]	1.7 [1.0; 3.1]	< 0.0001
Calcium (mmol/l)	2.4 [2.4; 2.5]	2.4 [2.4; 2.5]	n.s.
Phosphate (mmol/l)	1.2 [1.1; 1.3]	1.2 [1.1; 1.3]	n.s.
Creatinine (mg/dl)	0.9 [0.8; 1.0]	0.8 [0.7; 0.9]	< 0.05
GGT (U/l)	19 [15; 27]	17 [14; 24]	n.s.
C-reactive protein	0.17 [0.10; 0.28]	0.14 [0.07; 0.30]	n.s.
25-OH-vitamin D (mmol/l)	26 [18; 30]	25 [19; 34]	n.s.
PTH (pg/ml)	39 [34; 55]	38 [28; 50]	n.s.
AP (U/l)	69 [57; 79]	72 [59; 83]	n.s.
Osteocalcin (ng/ml)	19.6 [14.5; 28.1]	19.7 [16.9; 24.1]	n.s.
CTX (ng/ml)	0.30 [0.19; 0.53]	0.26 [0.18; 0.34]	n.s.

*GGT* gamma glutamyl transferase, *PTH* parathyroid hormone, *AP* alkaline phosphatase, *CTX* cross-linked-C-telopeptide of type I collagen

### Design and Quality Control of the osteomiR Analysis in Human Serum

The osteomiR® microRNA panel consists of 24 different assays (Supplemental Fig. 1). Of these, 19 are designed to detect emerging microRNA bone biomarkers per serum sample, which target multiple biological pathways that are relevant to bone metabolism (Supplemental Table 1). In-depth monitoring of sample quality and analytical variability is key for obtaining reliable circulating microRNA results [23]. In order to monitor analytical variability and sample quality, three spike-in controls, which are added prior to RNA extraction (RNA spike-in), reverse transcription (cDNA spike-in) and PCR (PCR spike-in), are detected per sample at positions 7, 16, and 20, respectively (Supplemental Fig. 1). Finally, the panel includes an assay for the red blood cell-enriched miR-451a (position 19, Supplemental Fig. 1) and miR-23a-3p as a reference for hemolysis (position 22, Supplemental Fig. 1) because of its low abundance in red blood cells.

**Fig. 1:**
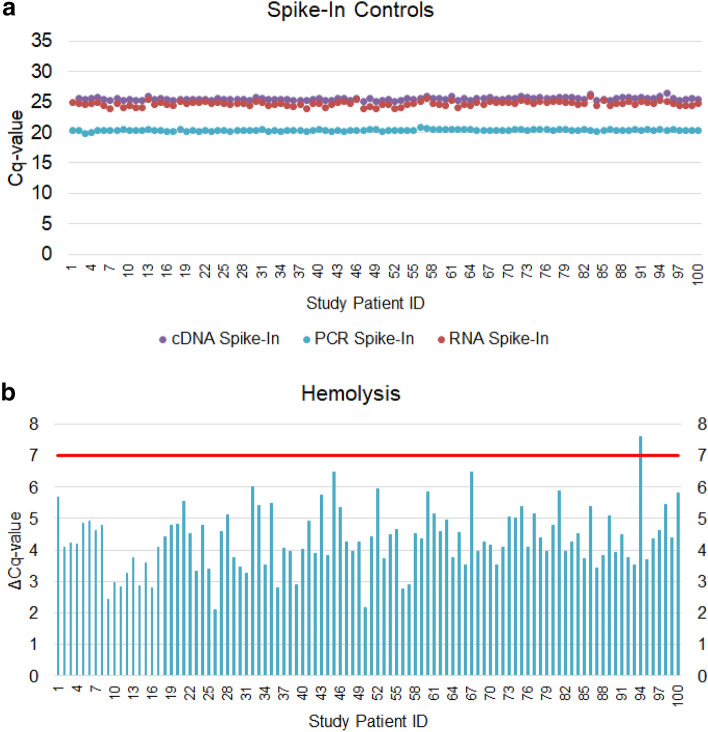
Quality control assessment of osteomiR analysis by RT-qPCR. **a** Spike-in controls added to the RNA extraction (red), cDNA synthesis (purple), and PCR reaction (turquoise) indicate the analytical variability. **b** The ratio (delta Cq-value) between miR-23a/miR-451a is an established indicator of hemolysis

By evaluating the quality controls in each sample, we observed low variability in spike-in levels across all 100 samples (Fig. 1a) and no signs of RT-qPCR inhibition. Hemolysis was assessed using the ratio of miR-23a/miR-451a [24] in all samples (Fig. 1b). We identified 1 out of 100 samples with elevated hemolysis (> 7), which was subsequently removed from the statistical analysis.

**Fig. 2:**
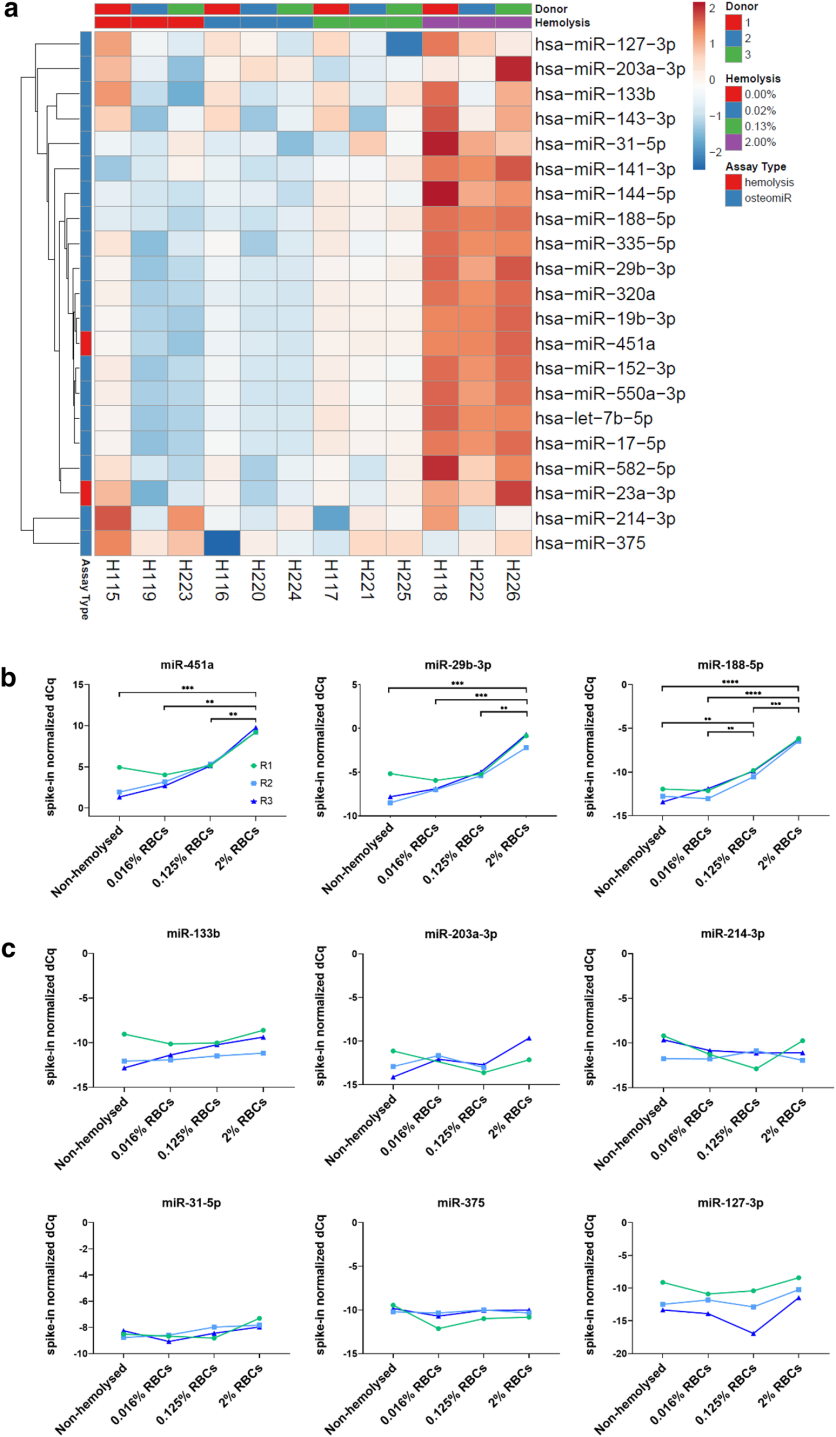
Effect of hemolysis on osteomiR levels in human serum. **a** Heatmap illustrating the increase in osteomiR levels between non-hemolytic (red group) to low (blue), moderate (green), and severely (purple) hemolytic samples. **b** miR-451a and two other exemplary osteomiRs that increase in serum with increasing hemolysis. **c** All six osteomiRs that are not sensitive towards hemolysis. One-way ANOVA with Tukey post hoc tests. *p < 0.05, **p < 0.01, ***p < 0.001, ****p < 0.0001

To systematically assess the interference between hemolysis and osteomiR analysis in human serum, we spiked increasing amounts of red blood cells (0.02, 0.13, and 2.00% v/v) to non-hemolytic serum, which resulted in a significant increase of free hemoglobin (OD414) as well as the hemolysis ratio (miR-23a/451a) at 0.013% RBC (Supplemental Fig. S2). We observed that 12 out of 19 osteomiRs clustered tightly with miR-451a (Fig. 2a) and showed a steady increase in concentration with increasing hemolysis (examples shown in Fig. 2b). OsteomiRs not affected by hemolysis were miR-133b, miR-203a-3p, miR-214-3p, miR-31-5p, miR-375, and miR-127-3p (Fig. 2c).

### OsteomiR Abundance in Human Serum and Correlation

**Fig. 3:**
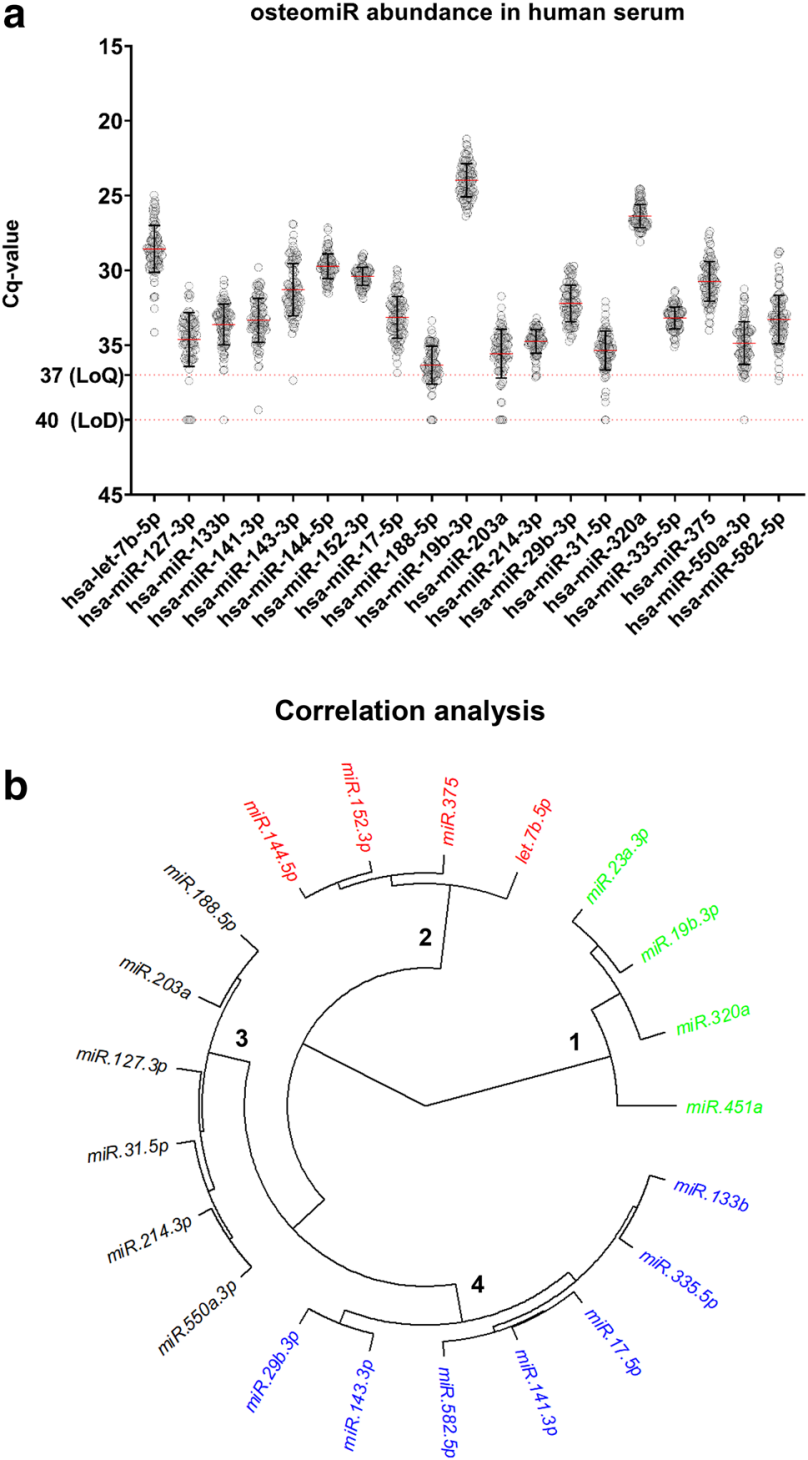
Abundance and correlation of osteomiR levels in serum of postmenopausal females. **a** Cq-values for 19 osteomiRs with respect to the limit of quantification (LoQ, Cq 37) and limit of detection (LoD, Cq 40) of the assay. **b** Unsupervised clustering of 19 osteomiRs based on Euclidean distance and ward.D2 clustering is shown as circular dendrogram. Four distinct clusters were identified and labeled 1–4

In the next step, the abundance of 19 osteomiRs in the entire cohort of 99 postmenopausal women was plotted using the raw data (Cq-values) from the RT-qPCR assay (Fig. 3a). We detected that eight miRNAs from the panel showed at least a few observations below the limit of quantification (LoQ) at a Cq-value 37. Five miRNAs (miR-127-3p, miR-188-5p, miR-203a, miR-31-5p, and miR-582-5p) were not detectable in up to 10% of the samples. It is known that circulating microRNA levels can be highly correlated, and thus we aimed to explore the similarity between osteomiR patterns in this cohort of 99 postmenopausal women using Pearson correlation. Figure 3b shows a circular dendrogram derived from hierarchical clustering analysis. Clustering identified at least 4 distinct clusters of miRNAs within the osteomiR panel.

### Diagnostic Performance of osteomiRs for Osteoporosis According to Who Criteria or History of Major Osteoporotic Fractures

**Fig. 4:**
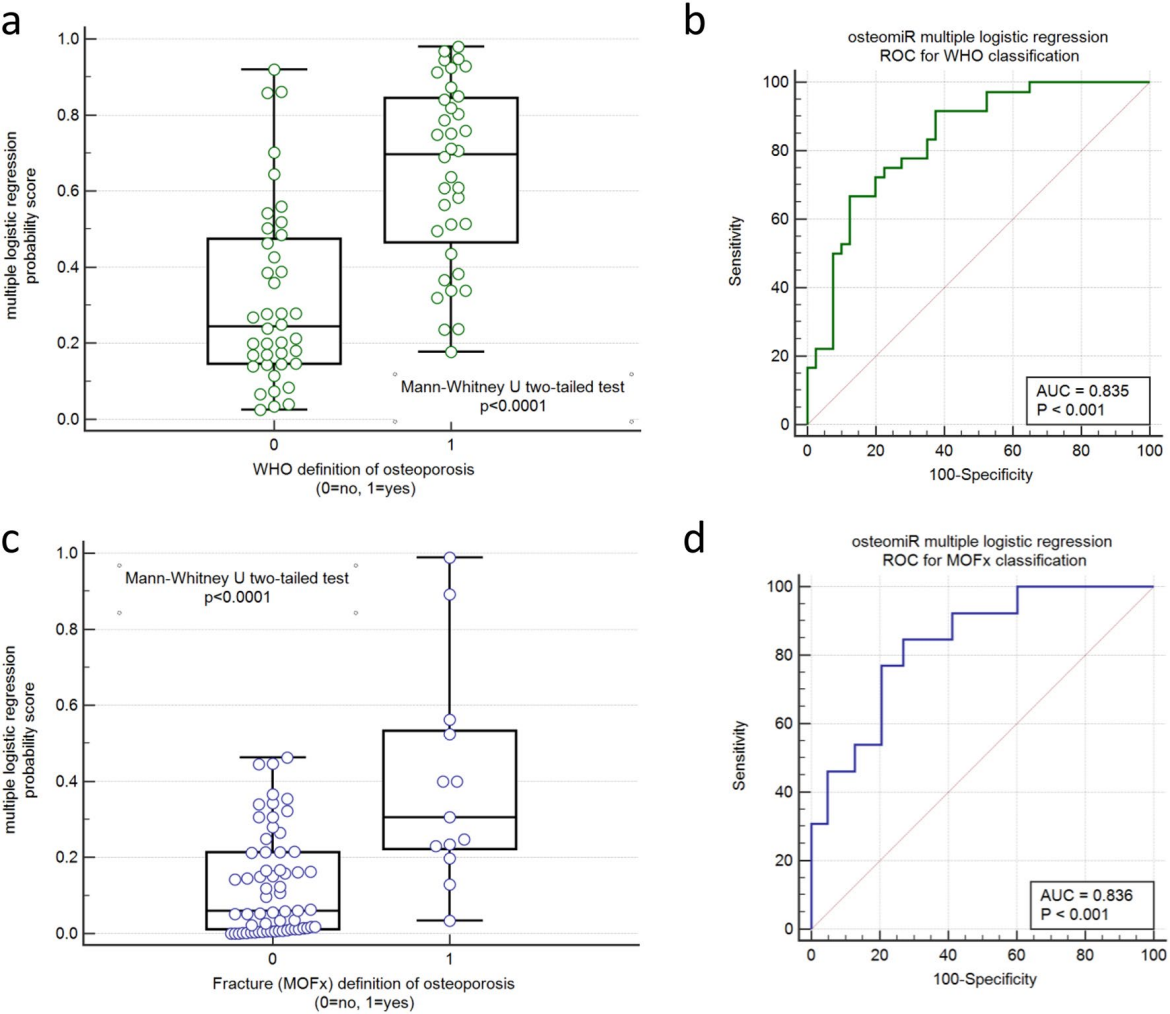
Diagnostic performance of osteomiRs based on the WHO and fracture-based definitions of osteoporosis. **a** The probability score (*p*-score) obtained from a multiple logistic regression model including 19 osteomiRs is shown for the WHO definition. **b** Classification performance of the *p*-score for WHO definition based on ROC analysis. **c** and **d** show the same analyses for the fracture-based definition of osteoporosis

We applied multiple logistic regression analysis to determine the relationship between osteomiR serum levels as independent variables with dichotomous definitions of osteoporosis (WHO and fracture-based). We found that combining 19 osteomiRs into a multivariate model can be used for the diagnosis osteoporosis based on the WHO (*p* < 0.0001, AUC = 0.830) and fracture-based definition (*p* < 0.0001, AUC = 0.834) with high accuracy (Fig. 4a–d).

Interestingly, different sets of microRNAs were found to have the strongest contribution to the model fit: miR-143-3p (OR 2.81), miR-188-5p (OR 0.54), miR-375 (OR 2.45), and miR-582-5p (OR 0.52) in the case of WHO definition, and miR-152-3p (OR 0.16), miR-203a-3p (OR 2.78), and miR-550a-3p (OR 0.11) in case of the MOFx definition. The independent contributions of osteomiRs to the models predicting WHO and fracture-based classification of osteoporosis are shown in Tables 3 and 4. miR-375 was identified as the strongest independent contributor to the WHO classification (*p* = 0.03), while miR-203a was identified as the strongest independent factor for the fracture-based classification (*p* = 0.05).

**Table 3:** Logistic regression based on WHO classification: independent osteomiR contributions (miRNAs with *p* < 0.1 are bold)

Variable	Coef	Std. err	Wald	*p*	OR	95% CI
hsa-let-7b-5p	0.46	0.36	1.67	0.20	1.58	0.7887–3.1721
hsa-miR-127-3p	− 0.14	0.24	0.37	0.54	0.87	0.5469–1.3739
hsa-miR-133b	− 0.26	0.33	0.65	0.42	0.77	0.4063–1.4545
hsa-miR-141-3p	− 0.73	0.56	1.74	0.19	0.48	0.1618–1.4277
hsa-miR-143-3p	1.03	0.57	3.31	**0.07**	2.81	0.9227–8.5742
hsa-miR-144-5p	− 1.01	0.63	2.55	0.11	0.36	0.1046–1.2598
hsa-miR-152-3p	− 0.78	0.84	0.87	0.35	0.46	0.0891–2.3546
hsa-miR-17-5p	− 0.12	0.53	0.05	0.82	0.88	0.3096–2.5191
hsa-miR-188-5p	− 0.62	0.30	4.11	**0.04**	0.54	0.2975–0.9795
hsa-miR-19b-3p	− 0.62	1.15	0.29	0.59	0.54	0.0559–5.1550
hsa-miR-203a	− 0.01	0.20	0.00	0.94	0.99	0.6661–1.4596
hsa-miR-214-3p	0.54	0.47	1.34	0.25	1.71	0.6873–4.2730
hsa-miR-29-3p	0.48	0.71	0.45	0.50	1.61	0.4022–6.4344
hsa-miR-31-5p	0.06	0.28	0.04	0.84	1.06	0.6070–1.8479
hsa-miR-320a	1.19	0.88	1.84	0.17	3.30	0.5886–18.4803
hsa-miR-335-5p	− 0.06	0.71	0.58	0.45	0.58	0.1455–2.3419
hsa-miR-375	0.90	0.41	4.86	**0.03**	2.45	1.1048–5.4264
hsa-miR-550a-3p	0.37	0.56	0.43	0.51	1.44	0.4832–4.3201
*hsa-miR-582-5p*	− 0.66	0.39	2.89	**0.09**	0.52	0.2432–1.1063

*Coef.* coefficient, *Std. err.* standard error

**Table 4:** Logistic regression based on fracture (MOFx) classification: independent osteomiR contributions (miRNAs with *p* < 0.15 are bold)

Variable	Coef	Std. err	Wald	*p*	OR	95% CI
hsa-let-7b-5p	0.59	0.57	1.11	0.29	1.81	0.5988–5.4868
hsa-miR-127-3p	0.01	0.39	0.00	0.99	1.01	0.4673–2.1656
hsa-miR-133b	− 0.50	0.59	0.71	0.40	0.61	0.1903–1.9345
hsa-miR-141-3p	0.45	0.59	0.59	0.44	1.57	0.4953–4.9952
hsa-miR-143-3p	0.46	0.97	0.23	0.63	1.59	0.2372–10.6490
hsa-miR-144-5p	0.12	0.75	0.02	0.88	1.13	0.2585–4.8974
hsa-miR-152-3p	− 1.86	1.22	2.34	**0.13**	0.16	0.0142–1.6913
hsa-miR-17-5p	0.00	0.68	0.00	1.00	1.00	0.2663–3.7868
hsa-miR-188-5p	− 0.16	0.45	0.13	0.72	0.85	0.3511–2.0585
hsa-miR-19b-3p	0.40	1.71	0.06	0.81	1.50	0.0526–42.6134
hsa-miR-203a	1.02	0.52	3.79	**0.05**	2.78	0.9934–7.7571
hsa-miR-214-3p	0.32	0.64	0.25	0.61	1.38	0.3923–4.8684
hsa-miR-29-3p	0.61	0.84	0.53	0.47	1.84	0.3571–9.5033
hsa-miR-31-5p	0.64	0.56	1.28	0.26	1.89	0.6288–5.6699
hsa-miR-320a	1.36	1.44	0.90	0.34	3.90	0.2328–65.2382
hsa-miR-335-5p	0.41	1.08	0.14	0.71	1.50	0.1817–12.4325
hsa-miR-375	0.07	0.48	0.02	0.89	1.07	0.4196–2.7358
hsa-miR-550a-3p	− 2.18	1.14	3.66	**0.06**	0.11	0.0121–1.0545
hsa-miR-582-5p	− 0.17	0.51	0.12	0.73	0.84	0.3063–2.3014

*Coef.* Coefficient, *Std. err.* standard error

## Discussion

This study is the first evaluating the combined diagnostic performance of osteomiR in postmenopausal women with osteoporosis and postmenopausal women with a history of MOFx. It shows that miRNAs of the osteomiR panel predict osteoporosis according to the WHO definition as well as the risk of MOFx with high accuracy. The classification performance for the WHO criteria is driven by miR-375 and the classification performance for risk of MOFx is driven by miR-203a-3p.

BMD measurement was performed to classify study participants according to the WHO definition as osteoporotic or non-osteoporotic. In subjects with osteoporosis, the risk of fracture according to the FRAX tool was significantly elevated. Additionally, their BTMs were significantly higher than in subjects without osteoporosis. As expected, stratifying patients according to the presence or absence of MOFx showed similar group-specific differences for BMD and the FRAX scores. Serum creatinine was higher in subjects with MOFx but was within the normal range in both groups. BTMs were similar in subjects with and without a MOFx. This is in line with a recent publication showing only a modest association between P1NP and CTX with future fracture risk after adjusting for BMD and clinical risk factors [25]. Serum levels of vitamin D did not show any group-specific differences but were generally low. Some study participants were supplemented with vitamin D. However, we do not think that this influences miRNA expression because even supplementation with high doses of vitamin D3 (20,000–40,000 IU per week) did not show a consistent effect on the mi RNA expression profile [26].

It is known that miRNAs are involved in several physiological and pathophysiological processes including those of bone metabolism. We used osteomiR, a panel of miRNAs, which has already proved high sensitivity and specificity [18]. In addition, miRNAs show a high stability in biofluids such as serum and are considered as promising new minimally invasive biomarkers for several pathological conditions including osteoporosis [6, 27]. However, hemolysis can be an important bias when analyzing levels of bone-related microRNAs in human serum [24, 28]. Here, we performed a systematic analysis of the effects of hemolysis on the detection of osteomiRs in human serum and showed that the majority of osteomiRs is sensitive towards this bias with the exception of miR-127-3p, miR-133b, miR-203a-3p, miR-214-3p, miR-31-5p, and miR-375. Thus, in order to achieve reliable results hemolysis needs to be controlled by either measuring the ratio of miR-23a/451a according to Blondal et al. [24] or optically using the absorbance at 414 nm. In this study, only one sample exhibited elevated hemolysis and had to be excluded from the analysis; measurement in 99 of 100 serum samples was successful. This underlines the practicability under real-life conditions.

We further evaluated the measurability of osteomiRs and observed that except for five miRNAs, which were below the LoQ or limit of detection (LoD) in 10% of the analyzed samples, the abundance of most osteomiRs was moderate to high in human serum.

The osteomiR signature has been defined on the basis of several studies, which explored associations between circulating microRNAs and different bone disease outcomes and musculoskeletal parameters [12, 15, 16, 18, 29–31]. The concept of osteomiRs is that they represent several independent factors that are associated with bone disease and risk of fractures. In order to validate that osteomiRs indeed represent a combination of independent factors, a high-quality dataset without systematic pre-analytical or analytical bias is required, such as the current data set for 99 postmenopausal women. Thus, we performed correlation analysis and observed that the osteomiR signature is composed of 4 clusters of miRNAs. Combinations of miRNAs from different clusters of the osteomiR were found to be important for the classification of osteoporosis according to the WHO as well as the MOFx classification.

MiRNAs with strong contribution in case of the WHO definition are miR-143-3p (cluster 4), miR-188-5p (cluster 3), miR-375 (cluster 2), and miR-582-5p (cluster 4). In knee osteoarthritis patients, miR-582-5p was significantly decreased in blood samples as well as in sclerotic subchondral bone; in cell culture, the expression of miR-582-5p was downregulated during osteogenic differentiation of mesenchymal stem cells [32]. The authors conclude that miR-582-5p is a negative regulator of osteoblast differentiation. Mesenchymal stem cells treated with serum obtained after a half-marathon promoted osteogenic differentiation corroborated by an upregulation of RUNX2 and a downregulation of miR-188-5p [33], a promotor of adipogenesis versus osteogenesis [34]. In fact, miR-188-5p has been shown to reduce the phosphatidylinositol-3-kinase (PI3K)/Akt pathway, a key regulatory pathway for bone formation [35]. The fact that these two miRNAs are negative regulators of bone formation appears to contradict the results of this study showing higher serum levels of miR-188-5p and miR-582-5p being associated with a lower risk of osteoporosis according to the WHO definition (ORs of 0.54 and 0.52, respectively). This discrepancy might be explained by the fact that those papers, which show miR-188-5p and miR-582-5p as negative regulators, do not directly relate to postmenopausal osteoporosis. MiR-143-3p has previously been shown to be highly associated with the osteogenesis of adipose-derived mesenchymal stem cells [36]. Osterix seems to be the direct target of miR-143 in suppressing osteogenic differentiation of MC3T3-E1 cells [37]. In miR-143 knockout mice, osteoblast differentiation was significantly inhibited [38]. All these data fit our results of high serum levels of miR-143-3p indicating a high risk of osteoporosis (OR 2.81).

The miRNA driving the classification performance for WHO is miR-375. Previous literature shows diverse effects of miR-375 on bone formation. Chen et al. [39] investigated human mesenchymal stem cells and found miR-375 enriched exosomes to improve osteogenic differentiation and to promote bone regeneration. However according to other studies, miR-375 negatively regulates RUNX2 expression, which is essential for osteoblastogenesis [40, 41]. Sun and co-authors [42] showed that miR-375 downregulates LRP5 and ß-catenin, indicating that it suppresses Wnt signaling pathways, which are essential for bone formation. These data demonstrating that miR-375 negatively modulates osteogenesis are in accordance with our results showing high serum levels of miR-375 indicating a high risk of osteoporosis (OR 2.45) and a previous clinical study. In 30 osteoporotic patients, miR-375 was higher than in 30 healthy subjects and its overexpression reduced the osteogenic effect of teriparatide, a drug frequently used in severe osteoporosis [41].

In case of the MOFx definition, two other miRNAs with strong contribution are miR-550a-3p (cluster 3) and miR-152-3p (cluster 2). Previous clinical data are diverse showing fragility fractures to be associated with either an upregulation, a downregulation, or no change of these miRNAs that could be related to different patient characteristics [15, 16, 18, 19]. MiR-550a-3p can be regarded as a marker of the dynamic processes of bone because in idiopathic osteoporosis it was found to be correlated to mineral apposition and bone formation rate [29]. Additionally, it was correlated with the bone turnover markers osteocalcin and CTX [18]. MiR-152-3p is supposed to be involved in signaling pathways regulating pluripotency of stem cells [43] and regulating the expression of WNT1OB, indicating an effect of miR-152-3p on the osteogenic differentiation of mesenchymal stem cells [44]. Remarkably, we detected several relevant miRNAs associated with bone formation but no association of miRNAs reflecting bone resorption.

The miRNA driving the classification performance for MOFx is miR-203a-3p (cluster 3). This is in line with previous experimental as well as clinical data. In postmenopausal women with and without type 2 diabetes, miR-203a was upregulated in subjects with history of fragility fracture [15]. Recently, it was also identified in patients with a mutation in PLS3 (manuscript submitted for publication), who develop severe early onset osteoporosis. A preclinical study showed an upregulation of miR-203a-3p in bone and serum of ovariectomized rats; osteoporosis-specific treatment reverted this increase [31]. Cell culture studies proved miR-203a-3p to be a suppressor of bone formation. In human osteoblastic cells, miR-203a negatively regulated osteoblastogenesis by upregulation of the downstream signal of bone morphogenetic protein (BMP)-2, distal-less homeobox 5 (Dlx5) which activates runt-related transcription factor 2 (Runx2) and osterix (Osc), both transcription factors for osteoblast differentiation [45]. Contrary, downregulation of miR-203a promotes osteoblastic differentiation of mesenchymal stem cells [46]. Taking all these studies into account miR-203a may serve as a diagnostic marker for osteoporosis and fragility fractures in postmenopausal women. This study showed a 2.78 times higher risk of fragility fractures for postmenopausal women with elevated miR-203a-3p.

Limitations of this study are twofold: first, we could only analyze data of 18 subjects with MOFx, leading to a relatively low power for the MOFx stratification compared to the WHO stratification. Second, no “control panel” of miRNAs, which were not previously known to be not associated to bone was analyzed. Thus, a negative control is missing. However, osteomiR is a signature of targeted miRNAs which has previously shown high sensitivity and specificity [16].

## Conclusions

This study proved the osteomiR panel to be a set of 19 emerging bone biomarkers, which together can be used as a fingerprint for osteoporosis based on the WHO criteria, but also show value in identifying individuals with a history of fractures. The panel includes a few tissue-enriched miRNAs; however, the majority is expressed by a variety of cell types including bone cells. Postmenopausal women with high serum levels of miR-375 had a high likelihood of being osteoporotic according to the WHO definition, which is most likely explained by negatively regulating the WNT signaling pathway. This study’s upregulation of miR-203a in subjects with a history of fragility fractures is in line with experimental data and one previous clinical study [15]. Thus, miR-203a may serve as a diagnostic marker for fragility fractures.

## Supplementary Information

Below is the link to the electronic supplementary material.

Electronic supplementary material 1 Fig. 1 OsteomiR® 384-well plate layout. A 384-well plate enabling the parallel analysis of 24 microRNAs in 16 samples. The panel includes 19 emerging bone biomarkers and 5 quality control assays. (PDF 265 kb)

Electronic supplementary material 2 Fig. 2 (a) Levels of free hemoglobin in serum samples spiked with increasing concentrations of red blood cells (RBCs) are shown. (b) Hemolysis ratio calculated on the basis of Cq(miR-23a) – Cq(451a), referred to as the hemolysis ratio. (c) Cq-values for miR-451a. (d) Cq-values for miR-23a-3p. One-way ANOVA with Tukey post hoc tests. *p<0.05, **p<0.01, ***p<0.001, ****p<0.0001. (PDF 294 kb)

Electronic supplementary material 1 (PDF 136 kb)

## Data Availability

microRNA RT-qPCR data together with all necessary metadata will be uploaded to a public data repository hosted by NCBI at https://www.ncbi.nlm.nih.gov/geo/.

## References

[1] Johnell O, Kanis JA (2004). An estimate of the worldwide prevalence, mortality and disability associated with hip fracture. Osteoporos Int.

[2] Schuit SC, van der Klift M, Weel AE, de Laet CE, Burger H, Seeman E (2004). Fracture incidence and association with bone mineral density in elderly men and women: the Rotterdam Study. Bone.

[3] Crandall CJ, Vasan S, LaCroix A, LeBoff MS, Cauley JA, Robbins JA (2018). Bone turnover markers are not associated with hip fracture risk: a case-control study in the Women’s Health Initiative. J Bone Min Res.

[4] Hackl M, Heilmeier U, Weilner S, Grillari J (2016). Circulating microRNAs as novel biomarkers for bone diseases. Complex signatures for multifactorial diseases?. Mol Cell Endocrinol.

[5] Zhao W, Shen G, Ren H, Liang D, Yu X, Zhang Z (2018). Therapeutic potential of microRNAs in osteoporosis function by regulating the biology of cells related to bone homeostasis. J Cell Physiol.

[6] Weilner S, Schraml E, Redl H, Grillari-Voglauer R, Grillari J (2013). Secretion of microvesicular miRNAs in cellular and organismal aging. Exp Gerontol.

[7] Wang Y, Li L, Moore BT, Peng XH, Fang X, Lappe JM (2012). MiR-133a in human circulating monocytes: a potential biomarker associated with postmenopausal osteoporosis. PLoS ONE.

[8] Li H, Wang Z, Fu Q, Zhang J (2014). Plasma miRNA levels correlate with sensitivity to bone mineral density in postmenopausal osteoporosis patients. Biomarkers.

[9] Cao Z, Moore BT, Wang Y, Peng XH, Lappe JM, Recker RR, Xiao P (2014). MiR-422a as a potential microRNA biomarker for postmenopausal osteoporosis. PLoS ONE.

[10] Meng J, Zhang D, Pan N, Sun N, Wang Q, Fan J (2015). Identification of miR-194-5p as a potential biomarker for postmenopausal osteoporosis. Peer J.

[11] Seeliger C, Karpinski K, Haug AT, Vester H, Schmitt A, Bauer JS, van Griensven, (2014). Five freely circulating miRNAs and bone tissue miRNAs are associated with osteoporotic fractures. J Bone Min Res.

[12] Weilner S, Skalicky S, Salzer B, Keider V, Wagner M, Hildner F (2015). Differentially circulating miRNAs after recent osteoporotic fractures can influence osteogenic differentiation. Bone.

[13] Panach L, Mifsut D, Tarin JJ, Cano A, Garcia-Perez MA (2015). Serum circulating microRNAs as biomarkers of osteoporotic fracture. Calcif Tissue Int.

[14] Garmilla-Ezquerra P, Sanudo C, Gelgado-Calle J, Perez-Nunez MI, Sumillera M, Riancho JA (2015). Analysis of the bone microRNome in osteoporotic fractures. Caclif Tissue Int.

[15] Heilmeier U, Hackl M, Skalicky S, Weilner S, Schroeder F, Vierlinger K (2016). Serum miRNA signatures are indicative of skeletal fractures in postmenopausal women with and without type 2 diabetes and influence osteogenic and adipogenic differentiation of adipose tissue-derived mesenchymal stem cells in vitro. J Bone Miner Res.

[16] Kocijan R, Muschitz C, Geiger E, Skalicky S, Baierl A, Dormann R (2016). Circulating microRNA signatures in patients with idiopathic and postmenopausal osteoporosis and fragility fractures. J Clin Endocrinol Metab.

[17] Yavropoulou MP, Anastasilakis AD, Makras P, Tsalikakis DG, Grammatiki M, Yovos JG (2017). Expression of micro RNAs that regulate bone turnover in the serum of postmenopausal women with low bone mass and vertebral fractures. Eur J Endocrinol.

[18] Zarecki P, Hackl M, Grillari J, Debono M, Eastell R (2020). Serum microRNAs as novel biomarkers for osteoporotic vertebral fractures. Bone.

[19] Ladang A, Beaudart C, LoCquet M, Reginster JY, Bruyere O, Cavalier E (2020). Evaluation of a panel of microRNAs that predicts fragility fracture risk: a pilot study. Calcif Tissue Int.

[20] Boschitsch EP, Durchschlag E, Dimai HP (2017). Age-related prevalence of osteoporosis and fragility fractures: real-world data from an Austrian menopause and osteoporosis clinic. Climacteric.

[21] Kanis JA, McCloskey EV, Johansson H, Strom O, Borgstrom F, Oden A (2008). Case finding for the management of osteoporosis with FRAX-assessment and intervention thresholds for the UK. Osteoporos Int.

[22] Metsalu T, Vilo J (2015). ClustVis: a web tool for visualizing clustering of multivariate data using Principal Component Analysis and heatmap. Nucleic Acids Res.

[23] Khan J, Lieberman JA, Lockwood CM (2017). Variability in, variability out: best practice recommendations to standardize pre-analytical variables in the detection of circulating and tissue microRNAs. Clin Chem Lab Med.

[24] Blondal T, Jensby Nielsen S, Baker A, Andreasen D, Mouritzen P, Wrang Teilum M, Dahlsveen IK (2013). Assessing sample and miRNA profile quality in serum and plasma or other biofluids. Methods.

[25] Tian A, Ma J, Feng K, Liu Z, Chen L, Jia H, Ma X (2019). Reference markers of bone turnover for prediction of fracture: a meta-analysis. J Orth Surg Res.

[26] Jorde R, Svartberg R, Joakimsen RM, Coucheron DH (2012). Plasma profile of microRNA after supplementation with high doses of vitamin D_3_ for 12 months. BMC Research Notes.

[27] Bottani M, Banfi G, Lombardi G (2020). The clinical potential of circulating miRNAs as biomarkers: present and future applications for diagnosis and prognosis of age-associated bone diseases. Biomolecules.

[28] Jacobsen N, Andreasen D, Mouritzen P (2011). Profiling microRNAs by real-time PCR. Methods Mol Biol.

[29] Weilner S, Schraml E, Wieser M, Messner P, Schneider K, Wassermann K (2016). Secreted microvesicular miR-31 inhibits osteogenic differentiation of mesenchymal stem cells. Aging Cell.

[30] Feichtinger X, Muschitz C, Heimel P, Baierl A, Fahrleitner-Pammer A, Redl H (2018). Bone-related circulating microRNAs miR-29b-3p, miR-550a-3p, and miR-324-3p and their association to bone microstructure and histomorphometry. Sci Rep.

[31] Kocijan R, Weigl M, Skalicky S, Geiger E, Ferguson J, Leinfellner G (2020). MicroRNA levels in bone and blood change during bisphosphonate and teriparatide therapy in an animal model of postmenopausal women. Bone.

[32] Wang P, Dong R, Wang B, Lou Z, Ying J, Xia C (2019). Genome-wide microRNA screening reveals miR-582-5p as a mesenchymal stem cell-specific microRNA in subchondral bone of the human knee joint. J Cell Physiol.

[33] Valenti MT, Deiana M, Cheri S, Dotta M, Zamboni F, Gabbiani D (2019). Physical exercise modulates miR-21-5p, miR-129-5p, miR-378-5p, and miR-188-5p expression in progenitor cells promoting osteogenesis. Cells.

[34] Li CJ, Cheng P, Liang MK, Chen YS, Lu Q, Wang JY (2015). MicroRNA-188 regulated age-related switch between osteoblast and adipocyte differentiation. J Clin Intest.

[35] Zhu W, Wu X, Yang B, Yao X, Xu P, Chen X (2019). miR-188-5p regulates proliferation and invasion via PI3K/Akt/MMP-2/9 signaling in keloids. Acta Biochim Biophys Sin.

[36] Jia B, Zhang Z, Qiu X, Chu H, Sun X, Zheng X, Zhao J, Li Q (2018). Analysis of the miRNA and mRNA involved in osteogenesis of adipose-derived mesenchymal stem cells. Exp Ther Med.

[37] Li E, Zhang J, Yuan T, Ma B (2014). MiR-143 suppresses osteogenic differentiation by targeting Osterix. Mol Cell Biochem.

[38] Wang R, Zhang H, Ding W, Fan Z, Ji B, Ding C (2020). miR-143 promotes angiogenesis and osteoblast differentiation by targeting HDAC7. Cell Death Dis.

[39] Chen S, Tang Y, Liu Y (2019). Exosomes derived from miR-375-overexpressing human adipose mesenchymal stem cells promote bone regeneration. Cell Prolif.

[40] Du F, Wu H, Zhou Z, Liu Y (2015). MicroRNA-375 inhibits osteogenic differentiation by targeting runt-related transcription factor 2. Exp Ther Med.

[41] Lei NB, Liang X, Wang P, Liu Q, Wang WG (2019). Teriparatide alleviates osteoporosis by promoting osteogenic differentiation of hMSCs via miR-375/RUNX2 axis. Eur Rev Med Pharmacol Sci.

[42] Sun T, Li CT, Xiong L, Ning Z, Leung F, Peng S, Lu WW (2017). miR-375-3p negatively regulates osteogenesis by targeting and decreasing the expression levels of LRP5 and β-catenin. PLoS ONE.

[43] Liu Y, Wang Y, Zhang Y, Liu Z, Xiang H, Peng X, Chen B, Jia G (2017). Screening for key pathways associated with the development of osteoporosis by bioinformatics analysis. Biomed Res Int.

[44] Vlachos IS, Zagganas K, Paraskevopoulou MD, Georgakilas G, Karagkouni D, Vergoulis T (2015). DIANA-miRPath v3.0: deciphering microRNA function with experimental support. Nucleic Acids Res.

[45] Laxman N, Mallmin H, Nilssn O, Kindmark A (2017). miR-203 and miR-320 regulate bone morphogenetic protein-2-induced osteoblast differentiation by targeting distal-less homeobox 5 (Dlx5). Genes.

[46] Xu X, Jiang H, Li X, Wu P, Liu J, Wang T, Zhou X, Xiong J, Li W (2017). Bioinformatics analysis on the differentiation of bone mesenchymal stem cells into osteoblasts and adipocytes. Mol Med Rep.

