# Supplementary figures and images for: Diagnostic Performance of a Panel of miRNAs (OsteomiR) for Osteoporosis in a Cohort of Postmenopausal Women
Source: Calcif Tissue Int. 2021 Jan 11;108(6):725–37. doi: 10.1007/s00223-020-00802-3 (PMC8166674; doi:10.1007/s00223-020-00802-3)

qPCR Amplification

384-well plate

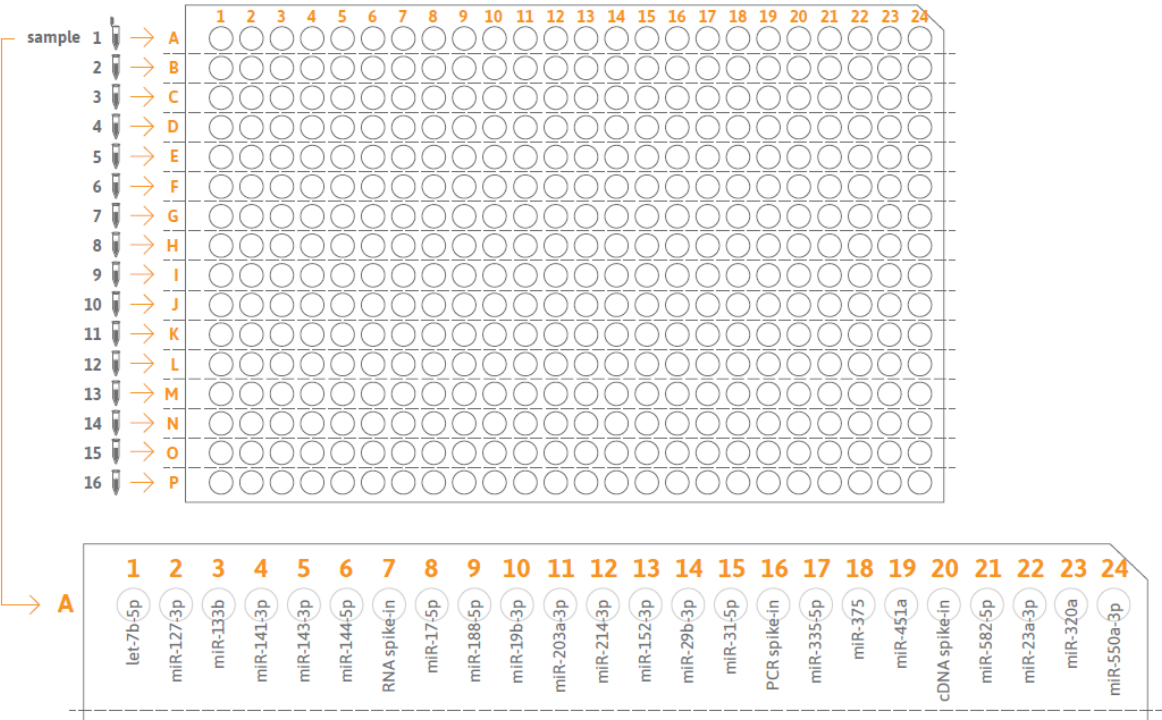

Supplement: Supplementary file 1 — Electronic supplementary material 1 Fig. 1 OsteomiR® 384-well plate layout. A 384-well plate enabling the parallel analysis of 24 microRNAs in 16 samples. The panel includes 19 emerging bone biomarkers and 5 quality control assays. (PDF 265 kb) [file 223_2020_802_MOESM1_ESM.pdf]

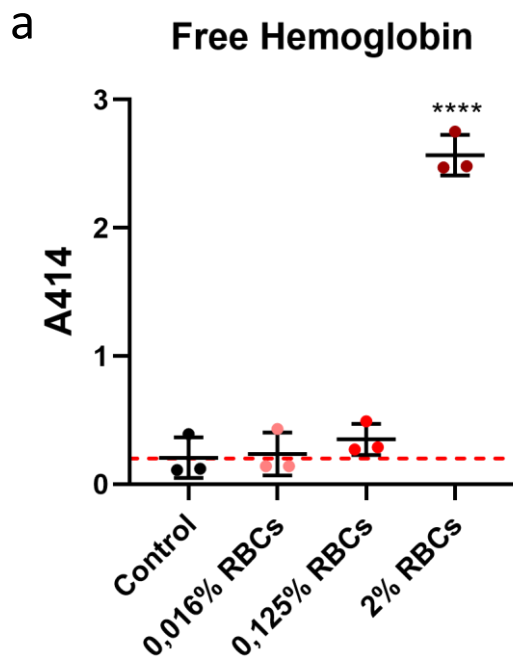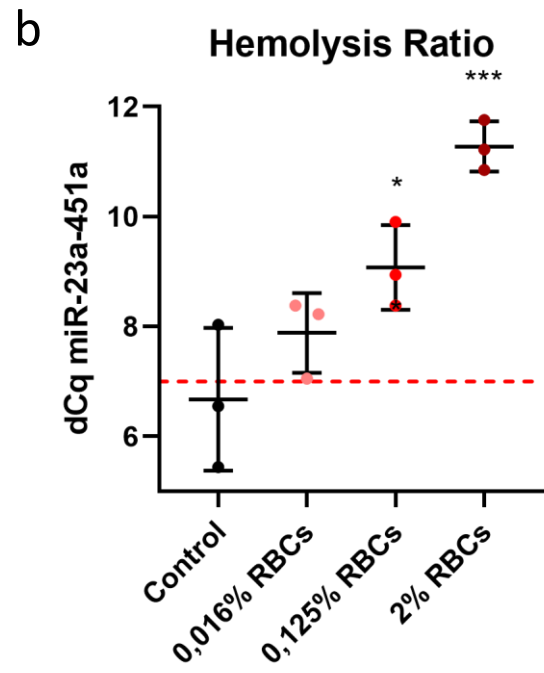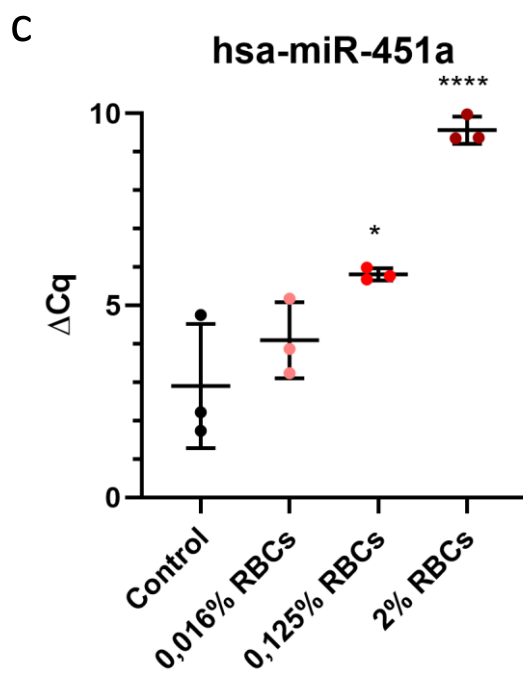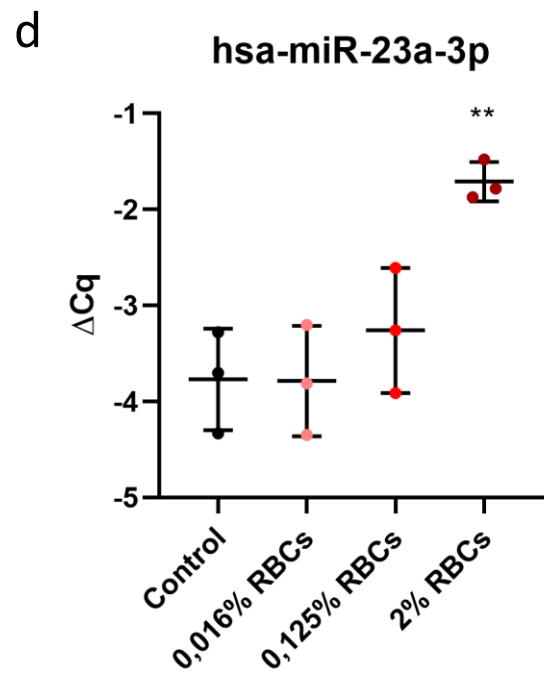

Supplement: Supplementary file 2 — Electronic supplementary material 2 Fig. 2 (a) Levels of free hemoglobin in serum samples spiked with increasing concentrations of red blood cells (RBCs) are shown. (b) Hemolysis ratio calculated on the basis of Cq(miR-23a) – Cq(451a), referred to as the hemolysis ratio. (c) Cq-values for miR-451a. (d) Cq-values for miR-23a-3p. One-way ANOVA with Tukey post hoc tests. *p<0.05, **p<0.01, ***p<0.001, ****p<0.0001. (PDF 294 kb) [file 223_2020_802_MOESM2_ESM.pdf]
