# Supplementary material for: Diagnostic Performance of a Panel of miRNAs (OsteomiR) for Osteoporosis in a Cohort of Postmenopausal Women
Source: Calcif Tissue Int. 2021 Jan 11;108(6):725–37. doi: 10.1007/s00223-020-00802-3 (PMC8166674; doi:10.1007/s00223-020-00802-3)
Supplement: Supplementary file 3 — Electronic supplementary material 1 (PDF 136 kb) [file 223_2020_802_MOESM3_ESM.pdf]

Diagnostic performance of a panel of miRNAs (OsteomiR) for osteoporosis in a cohort of postmenopausal women

microRNAs in postmenopausal osteoporosis

K. Kerschan-Schindl[1], M. Hackl[2,3], E. Boschitsch[4], U. Föger-Samwald[5], O. Nägele[4], S. Skalicky[2], M. Weigl[2,3], J. Grillari[3, 6, 7], P. Pietschmann[5]

[1] Department of Physical Medicine, Rehabilitation and Occupational Medicine, Medical University of Vienna, Vienna, Austria

[2] TAmiRNA GmbH, Vienna Austria

[3] Austrian Cluster for Tissue Regeneration

[4] KLIMAX Menopause and Osteoporosis Clinic, Vienna, Austria

[5] Institute of Pathophysiology and Allergy Research, Center for Pathophysiology, Infectiology and Immunology, Medical University of Vienna, Vienna, Austria

[6] Christian Doppler Laboratory for Biotechnology of Skin Aging, Department of Biotechnology, BOKU - University of Natural Resources and Life Sciences Vienna

[7] Ludwig Boltzmann Institute for Experimental and Clinical Traumatology, 1220 Vienna, Austria

Corresponding author:

Katharina Kerschan-Schindl

Department of Physical Medicine, Rehabilitation and Occupational Therapy, Medical University of Vienna, Austria

E-Mail: Katharina.Kerschan-Schindl@meduniwien.ac.at

Tel: 004314040043330

Fax: 004314040052800

ORCID: 0000-0002-1128-7532

Supplemental Table 1 Summary of current knowledge about the biological function of osteomiRs in the context of bone diseases and bone metabolism.

**miRNA ID**	**Bone turnover**	**Microstructure and histo-morphometry**	**Osteoporosis & other bone diseases**	**Bone loss & treatment response**	**Calcification**	**Therapeutic activity**	**Mechanism of action (selected references)**
let-7b-5p	x	x	x				VEGF, HMGA2 [15]
miR-127-3p			x				S1PR3 [18]
miR-133b	x		x	x			Runx2/FOXC1 [15, 18]
miR-141-3p			x		x		WNT [13]
miR-143-3p	x						17ß-estradiol, osterix[12, 18]
miR-144-5p	x						RANK [19]
miR-152-3p			x				[16]
miR-17-5p				x	X		Smad5, BMP2[19]
miR-188-5p			x	x		x	PPARγ via HDAC9/RICTOR [18]
miR-19b-3p	x		x	x			[16, 18]
miR-203a			x	x	x		Runx2, Dlx5, 17ß-estradiol [15, 16]

miR-214-3p					x	x	AT4 [18]
miR-29b-3p	x	x	x		x		HDC4, TGFß3, CTNNBIP1 [29]
miR-31-5p			x	x	X	x	WNT via FZD3 [19]
miR-320a			x		x		HOXA10 [12]
miR-335-5p	x	x	x	x	x		WNT via Dkk1 [16, 18]
miR-375			x		x	x	WNT via LP5 and ß-catenin [15, 18, 41]
miR-550a-3p	x	x	x		x		[15, 16, 18, 29]
miR-582-5p			x				[19]
